# Radiographic measurement of the posterior tibial slope in normal Chinese adults: a retrospective cohort study

**DOI:** 10.1186/s12891-022-05319-4

**Published:** 2022-04-26

**Authors:** Yong Chen, Jianping Ding, Siyu Dai, Jiao Yang, Mengke Wang, Tian Tian, Xiaolong Deng, Boyi Li, Guohua Cheng, Jie Liu

**Affiliations:** 1 Department of Radiology, The Affiliated Hospital of Hangzhou Normal University, Wenzhoulu, Gongshu District, Hangzhou City, 310000 Zhejiang Province China; 2 School of Clinical Medicine, Hangzhou Normal University, Hangzhou, China; 3 Department of Radiology, the Second Affiliated Hospital of Xi’an Medical University, Xi’an, China; 4 The Second People’s Hospital of Chun’an County, Hangzhou, China; 5 Hangzhou Jianpei Technology Co., Ltd, Hangzhou, China

**Keywords:** Anterior cruciate ligament, Arthroplasty, Chinese, Normal value, Posterior tibial slope

## Abstract

**Background:**

Measurement of the posterior tibial slope (PTS) angle has important applications in total knee replacement surgery, high tibial osteotomy, and anterior cruciate ligament reconstruction. This study aimed to determine the mean PTS of knee joints in healthy Chinese adults, and provide data to guide knee surgery in China.

**Methods:**

A retrospective analysis of 1257 (*n* = 1233, 50.4% male) plain X-ray films of participants aged 25–59 years was performed. The picture archiving and communication system was used for PTS measurement. The PTS was defined as the angle between the vertical line of the tangent of the anterior tibial cortex of the proximal tibia, and the tangent line of the tibial cortex. Two imaging physicians conducted the PTS measurements independently, and both the inter- and intraclass correlation coefficients (ICCs) were calculated.

**Results:**

The mean PTS value was 7.68 ± 3.84° (range: 0–21°). The left PTS was significantly smaller in males than in females (7.22 ± 3.89 vs 8.05 ± 3.60; *P* = 0.005). Additionally, the PTS in participants aged 25–29 years was significantly larger than that in the other age groups (Left side: 8.64 ± 3.73 vs 6.92 ± 3.42, 7.42 ± 3.75, 7.53 ± 3.98; *P* <  0.001 and Right side: 8.68 ± 3.84 vs 7.48 ± 4.21, 7.13 ± 3.64, 7.66 ± 3.80; *P* = 0.004). There were no significant differences in PTS between the left and right sides. Two-way analysis of variance suggested that the differences in PTS between age groups were not affected by sex. The interobserver ICC was 0.91 (95% confidence interval [CI]: 0.85–0.94), and the intraobserver ICC was 0.90 (95% CI: 0.82–0.94).

**Conclusions:**

This study demonstrated that there were significant differences in PTS based on sex and age, highlighting the need to provide individualized treatment for knee surgery. It provided valuable information regarding the normal PTS values in Chinese adults and presented regionalised data to guide knee surgery.

## Background

The posterior tibial slope (PTS) is the angle formed between the vertical line of the tibial anatomical axis and the tibial plateau tangent. It reflects the tilt of the tibial plateau and plays an important role in knee joint stability and biomechanics [1–8]. Measurement of the PTS has important applications in total knee arthroplasty (TKA), high tibial osteotomy (HTO), and anterior cruciate ligament (ACL) reconstruction surgery [3].

Changes in the PTS can lead to a series of significant clinical symptoms. For instance, increases in the PTS will cause the sagittal line of force to shift from the front to the back of the tibia and the contact point between the tibia and the femoral condyle to move backward. This will increase the pressure on the back of the tibial plateau, and the increase in the distance between the femoral and tibial stops of the ACL will lead to an increase in the tension on the ACL, which can cause anterior and posterior instability of the knee joint, enhancing the risk of ACL injury [1, 9–13]. Conversely, a decrease in the PTS will cause the sagittal force line to move forward, increasing the stress on the front of the tibial plateau. This will reduce the distance between the femoral and tibial stops of the posterior cruciate ligament (PCL), resulting in PCL tension [14].

After TKA, if the PTS is too large, it will increase the pressure on the back of the tibial prosthesis and the wear on the back of the prosthesis, promoting wear on the polyethylene prosthesis during joint movement, and resulting in aseptic loosening. Conversely, if the PTS decreases, the pressure will move forward, increasing the pressure on the front of the tibia, causing the tibial prosthesis to sink [5, 7]. Additionally, the increased PTS will change the positional relationship between the tibia and the femur. Therefore, when the knee is in flexion, the required knee extension force is reduced [7, 15–17].

Several previous studies have measured PTS, but their methodologies and the measurement equipment used varied markedly. Furthermore, PTS values differ markedly based on ethnicity [4, 18–21]. Moreover, there is controversy as to whether PTS is associated with sex and age in different populations [4, 22, 23]. Nevertheless, there is a paucity of research on the PTS in the Chinese population and therefore, the average PTS value is unknown in this population.

Thus, the current study aimed to determine the mean PTS of knee joints in healthy Chinese adults. Additionally, this study aimed to explore whether there was a correlation between PTS and sex, age, and the PTS side. The data from this study could help provide guidance for knee surgery and prosthesis manufacturers.

## Methods

### Study design and participants

Using the Picture Archiving and Communication System (PACS), we retrospectively analysed 13,392 patients who underwent knee X-ray examination in the Affiliated Hospital of Hangzhou Normal University in Zhejiang Province, China, from January 2017 to April 2021, due to knee injury, discomfort, physical examination, or re-examination [6, 18, 24–30]. Previous studies have shown that the age range of closure time of the knee epiphysis varies greatly across different races and regions. According to a study by Aljuaid et al. [30–33], the closure time of knee epiphysis varies from 14 to 24 years of age, and there are differences between the sexes. To reduce error in judgement of the failure of epiphyseal closure, due to late measurement, the latest age for human epiphyseal closure is defined as 25 years. At the same time, different degrees of osteohyperplasia are common in the knee joints of people aged ≥60. The occurrence of osteohyperplasia will seriously affect PTS measurement, causing an erroneous increase in PTS, which has also been confirmed in preliminary experiments, consistent with the report of Zhang et al. [5, 19, 24, 34]. Hence, the maximum age for inclusion was determined to be 59 years. Thus, the inclusion criteria for this study were as follows: (1) age 25–59 years; (2) no joint deformity; (3) no history of congenital disease, developmental deformity or related trauma with fracture, tumours, rheumatism, or inflammation; (4) clear X-ray images, with only patients with true lateral knee radiographs being included. The true lateral radiographs have a good overlap of the internal and external condyles of the femur. Lateral radiographs were excluded if there was > 5 mm malalignment of the posterior condyles [15, 18, 35–38]. The exclusion criteria were as follows: (1) non-Chinese ethnicity; (2) unclosed epiphysis; (3) obvious bone degeneration or osteoarthritis of the knee joint; (4) displaced fracture around the knee and/or a history of knee surgery.

Based on these criteria, we excluded 2672 patients aged > 59 years and 3094 patients aged < 25 years. The X-ray films were not of sufficient quality for measurement in 3240 cases. Additionally, we excluded 3153 cases of osteoarthropathy, soft tissue diseases after knee joint operation, osteoarthritis, fracture, and bone tumor. Eventually, 1233 subjects (1257 knees) were included in the study, all of whom had normal knee X-rays.

### Digital radiography

We used a Definium 6000 (GE Healthcare, Chicago, IL) to take lateral view images of the knee joints. Criteria for qualifying images were as follows: The images included the distal femur, knee joint space, and proximal tibia and fibula. The knee joint space was at the centre of the image, and the femoral internal and external condyles overlapped well. Malalignment of the posterior condyles was < 5 mm [37]. The patella was displayed laterally, the gap between the patella and the femur was clearly displayed, and there was no bilateral joint on the articular surface. There was limited overlap between the femoral condyle and the tibial plateau articular surface. The soft tissues were also clearly displayed.

### Quantitative anatomic measurements

**Fig. 1:**
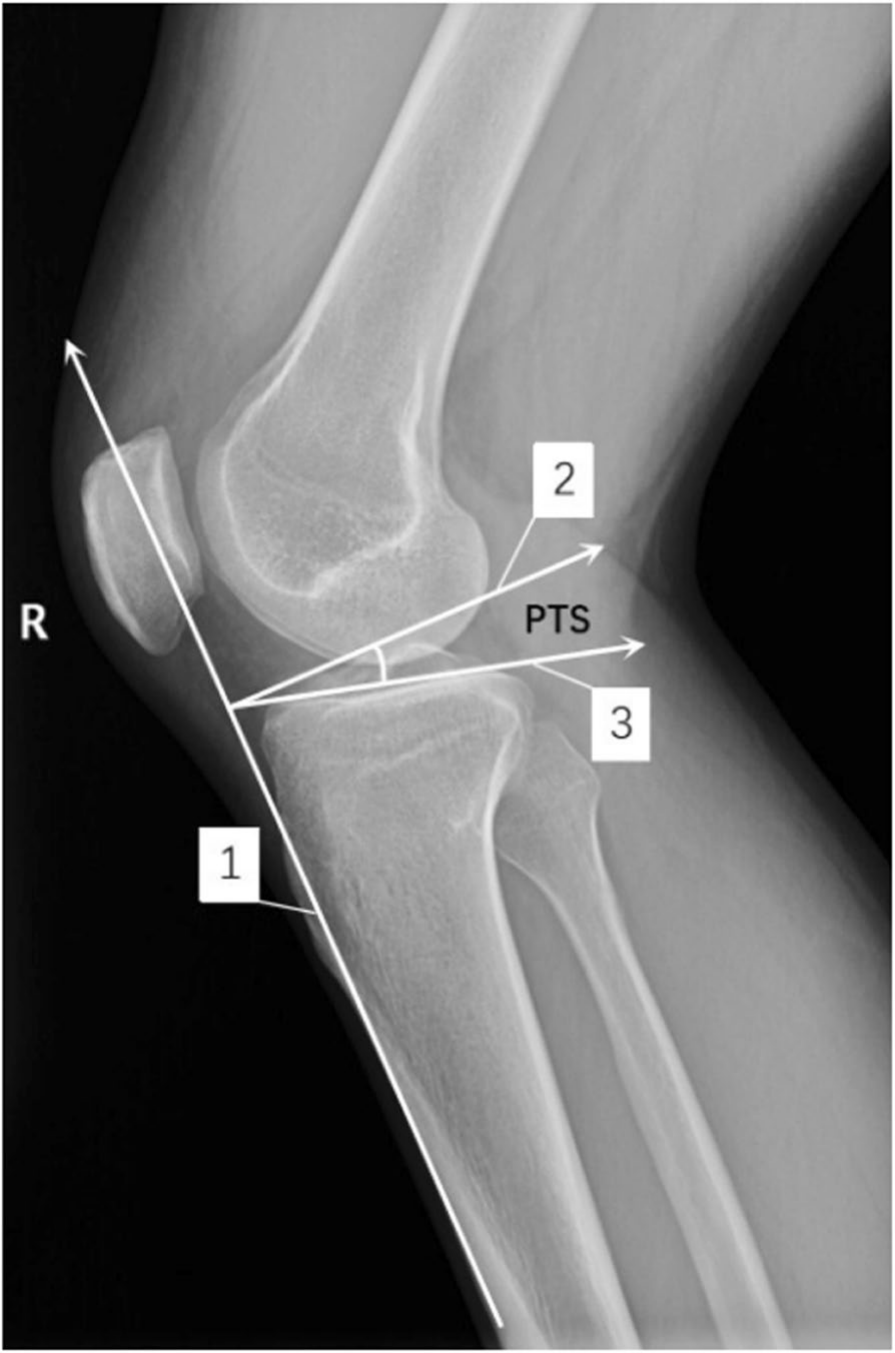
The measurement method of posterior tibial slope (PTS)^*^. ^*^X-ray lateral inspection: line 1 is the tangent line of the proximal tibia on the anterior cortex surface, line 2 is the perpendicular to line 1, and line 3 is the tangent line of the tibial plateau

The PTS was observed and measured using GE Centricity PACS software. The anterior tibial cortex method was used. First, line 1 was made tangential to the anterior cortex of the upper segment of the tibia on the lateral X-ray image, to represent the long axis of the tibia. Then, line 2 was drawn perpendicular to tangential line 1. Finally, line 3 was drawn tangential to the tibial plateau. The angles formed by lines 2 and 3 represent the PTS (Fig. 1) [4].

### Reliability analysis

The PTS was measured independently by two imaging physicians (physicians engaged in musculoskeletal diagnoses), and correlation analyses between and within groups were performed. To evaluate interobserver reliability, reader A measured all the data (*n* = 1257) and reader B randomly selected 80 cases for independent measurement; the measurement process used a double-blind method. To assess intraobserver reliability, after 4 weeks, reader A randomly selected 80 cases from all the measured data for re-measurement [18].

### Statistical analysis

SPSS software (ver. 25.0; IBM, Armonk, NY) was used for statistical analysis. T-tests were used to compare the PTS between the different PST sides and sexes. One-way analysis of variance (ANOVA) was used to compare the PTS between the different age groups. Two-way ANOVA was used to investigate the interaction of age and sex with PTS. The intraclass correlation coefficients (ICCs) together with their 95% confidence intervals (CIs) were used to evaluate the inter- and intraobserver correlations, with 0.75 ≤ ICC ≤ 1.00 considered to indicate good agreement. *P*-values < 0.05 were considered statistically significant.

### Ethics

Ethical approval was obtained from the institutional review board of the Affiliated Hospital of Hangzhou Normal University (Reference number: 2021(E2)-KS-074). The need to obtain informed consent from patients was waived due to the use of anonymized patient data and the retrospective study design.

## Results

**Fig. 2:**
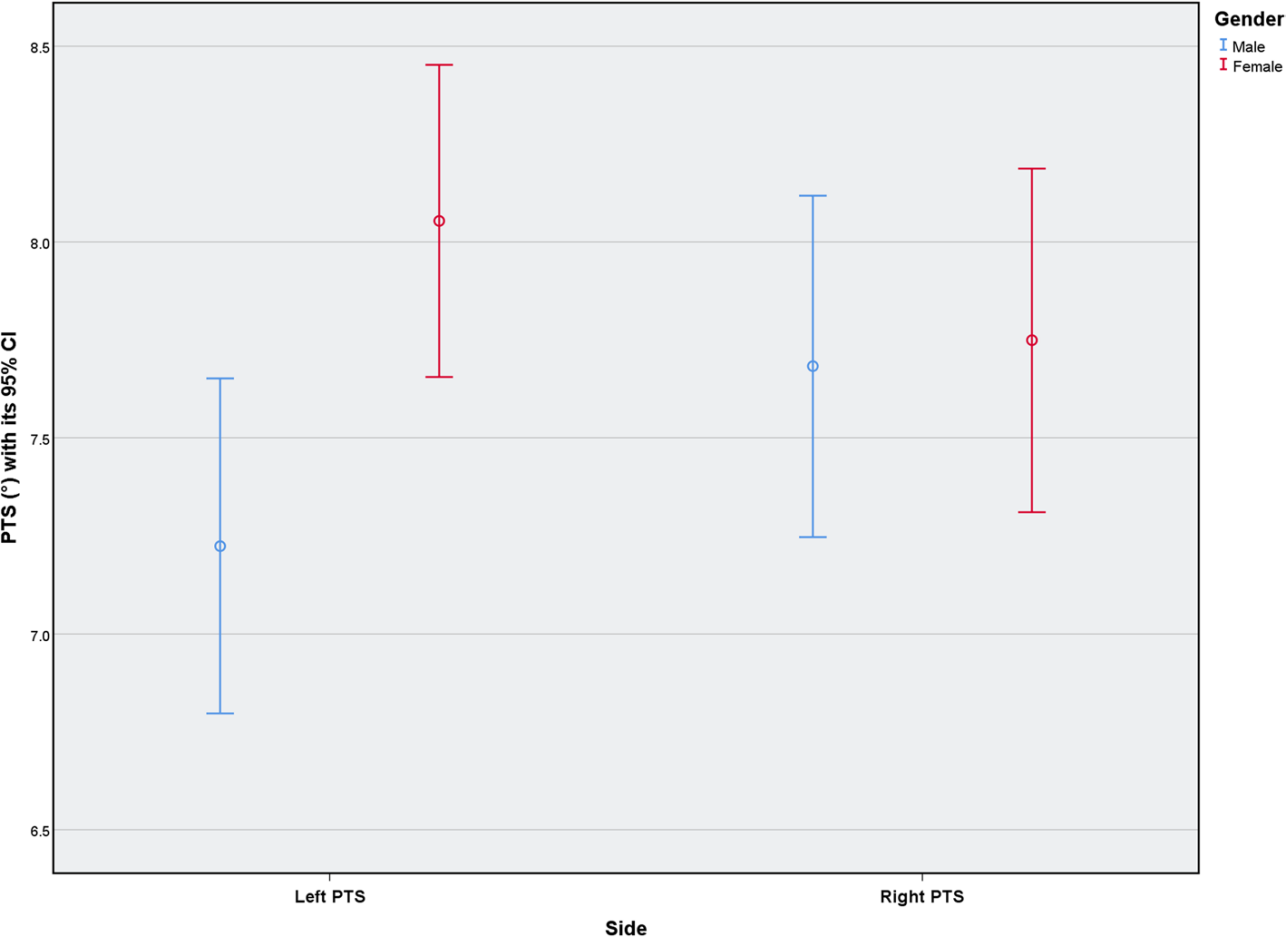
Distribution of posterior tibial slope (PTS) (°) by genders

**Fig. 3:**
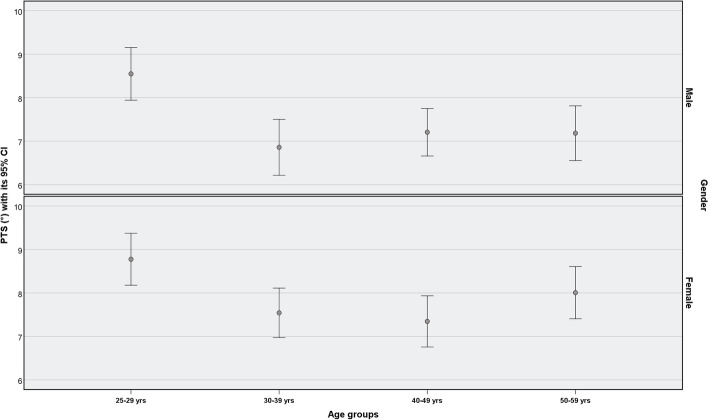
Distribution of posterior tibial slope PTS (°) by genders and age groups

A total of 1233 subjects (50.4% males) were included in this study. They were divided into four age groups: group A comprised 25–29-year-olds (*n* = 306, 24.8%), group B comprised 30–39-year-olds (*n* = 306, 24.8%), group C comprised 40–49-year-olds (*n* = 320, 26.0%), and group D comprised 50–59-year-olds (*n* = 301, 24.4%) (Table 1). In 637 and 620 cases of the left and right knees, respectively, the PTS was 7.64 ± 3.77° (range: 0–20°) and 7.72 ± 3.91 (range: 0–21°), respectively (Table 2). Paired-samples *t*-tests showed that there was no significant difference in PTS between the left and right sides (*P* > 0.05) (Table 2). However, the average PTS value on the left side was significantly smaller in males than in females (7.22° vs. 8.05°, *P* = 0.005) (Table 3, Fig. 2). One-way ANOVA showed that there were significant differences in PTS based on age-grouping (left PTS: *P* <  0.001; right PTS: *P* = 0.004). PTS of the 25–29-year age-group was significantly greater than that of the other age groups. The 30–39- and 40–49-year age-groups had smaller average PTSs, while the 50–59-year age-group had a slightly larger mean PTS than did the 30–39- and 40–49-year age-groups (Table 4, Fig. 3). Two-way ANOVA showed that the difference in PTS between the age groups was independent of sex (*P* > 0.05) (Table 5). PTS with interobserver ICCs of 0.91 (95%CI: 0.85–0.94) and intraobserver ICCs of 0.90 (95%CI: 0.82–0.94) were considered to show high inter- and intraobserver reliability.

**Table 1:** Demographics of the study participants (*N* = 1233)

Characteristics	N (%)
Sex	
Male	621 (50.4)
Female	612 (49.6)
Age, yrs.	
Group A (25–29)	306 (24.8)
Group B (30–39)	306 (24.8)
Group C (40–49)	320 (26.0)
Group D (50–59)	301 (24.4)

**Table 2:** Distribution of the mean PTS (°)

PTS (°)	N	Mean	SD	Range	T value^*^	*P* value^*^	Mean ± SD for all
Left side	637	7.64	3.77	0–20	−0.616	0.54	7.68 ± 3.84
Right side	620	7.72	3.91	0–21			

^*^By paired t test. *N* = 613

**Table 3:** PTS (°) characteristics by sex

	Male			Female			T value	*P* value
	N	Mean	SD	N	Mean	SD		
Left side	321	7.22	3.89	316	8.05	3.60	−2.79	**0.005**
Right side	309	7.68	3.89	311	7.75	3.93	−0.21	0.83

**Table 4:** PTS (°) characteristics by age groups

	Group A (25–29)		Group B (30–39)		Group C (40–49)		Group D (50–59)		F value	*P* value
	Mean (N)	SD	Mean (N)	SD	Mean (N)	SD	Mean (N)	SD		
Left side	8.64 (163)	3.73	6.92 (155)	3.42	7.42 (165)	3.75	7.53 (154)	3.98	6.09	**< 0.001**
Right side	8.68 (145)	3.84	7.48 (155)	4.21	7.13 (165)	3.64	7.66 (155)	3.80	4.45	**0.004**

**Table 5:** PTS (°) characteristics by sex and age groups

Age	Male				Female				F value			*P* value		
	Group A	Group B	Group C	Group D	Group A	Group B	Group C	Group D	sex	age	sex*age	sex	age	sex*age
Left side	8.36 ± 3.82 *N* = 88	6.45 ± 3.62 *N* = 76	6.85 ± 3.72 *N* = 75	7.06 ± 4.14 *N* = 82	8.96 ± 3.62 *N* = 75	7.37 ± 3.17 *N* = 79	7.89 ± 3.73 *N* = 90	8.07 ± 3.74 *N* = 72	9.12	6.42	0.12	0.003	**< 0.001**	0.95
Right side	8.78 ± 3.89 *N* = 69	7.25 ± 4.42 *N* = 79	7.50 ± 3.41 *N* = 90	7.32 ± 3.70 *N* = 71	8.59 ± 3.82 *N* = 76	7.72 ± 3.99 N = 76	6.69 ± 3.87 *N* = 75	7.95 ± 3.88 *N* = 84	0.007	4.62	1.15	0.94	**0.003**	0.33

## Discussion

The present study determined the mean PTS of knee joints in healthy Chinese adults, with a view to providing a guide for knee surgery in China. We demonstrated that PTS differed significantly based on sex and age, highlighting the need for individualized knee surgery. As we present PTS data for Chinese adults, our data can be used to guide knee surgery in China.

The stability of the knee joint involves dynamic and static components. The surrounding muscle tissue provides dynamic stability, whereas the bone structure, joint capsule, and attached ligaments provide static stability. The size of the PTS directly affects the position of the sagittal force line of the lower limbs, which in turn affects the stability of the knee joint [18]. The PTS is defined as the angle formed by the vertical line of the tibial anatomical axis and the tibial plateau tangent [1]. The PTS can be measured using various methods, which include X-ray, computed tomography (CT), and magnetic resonance imaging (MRI). CT and MRI have the advantage that they can accurately measure the inner tibia and lateral posterior angle. However, their disadvantages, which include low equipment penetration, long inspection times, high costs, the need for patient cooperation, and the small scanning range, make it difficult to determine the anatomical axis of the tibia, thus requiring standard methods for interpretation. These methods are used less commonly in clinical practice. The advantages of X-rays are the high equipment penetration rates, short inspection times, low cost, lower radiation dose than that in CT [36], fewer contraindications than those of MRI [35], large irradiation range, and ease of ability to determine the anatomical axis of the tibia. Additionally, X-rays allow clinicians to complete the measurements independently, and can be used for pre- and post-evaluations. The disadvantage in using X-rays for measurement is the difficulty in distinguishing the medial and lateral plateaus of the tibia, as the lateral image requires an overlap of the medial and lateral platforms [39]. Therefore, X-ray measurements lack consistency as compared to that of CT and MRI [35].

At the same time, there are many methods that can be used to obtain X-ray measurements, including those using the anterior tibial cortex (ATC), posterior tibial cortex (PTC), tibial proximal anatomical axis (TPAA), tibial shaft anatomical axis, fibular proximal anatomical axis, and fibular shaft axis [12, 40–42]. Although the various methods differ (Table 6), the PTS values derived from them correlate [6]. At present, the clinically most widely adopted methods are those using the TPAA and the ATC. The extramedullary positioning method is often used in knee surgery, during which the positioning rod is parallel to the ATC, and then the PTS is measured with reference to the positioning rod. Thus, the PTS value measured using the ATC method is often referenced in preoperative planning. The current study employed the ATC method.

**Table 6:** Comparison with previous similar research

Author	Year	Sample size	Sample	Country/ region	Age	Sex	Measurement method	Measurement principle	PTS	
									Range	Mean ± SD
The current research	2021	1233	Healthy adults	China	25–59	Male and Female	X-ray	ATC	0–21	7.68 ± 3.84
Bao et al. [40]	2021	80	Healthy adults	China	20–45	Male and Female	CT	TPAA	Medial: 0.05–12.04 Lateral: −0.30-14.99	Medial: 6.78 Lateral: 6.11
Kacmaz IE et al. [18]	2020	1024	Healthy adults	Turkey	18–92	Male and Female	X-ray	TPAA	2.1–18.7	8.36 ± 3.3
Mısır et al. [20]	2018	1000	Healthy adults	Turkey	18–50	Male and Female	MRI	TPAA		4.9 ± 1.9
Han et al. [23]	2016	535	non-arthritic knees of adults	Korean	20–79	Male and Female	MRI	TPAA		Medial: 6.82 ± 1.81Lateral: 6.09 ± 1.73
Zhang et al. [21]	2014	80	Healthy adults	China	20–45	Male and Female	CT	TPAA		8.4 ± 3.1
			healthy adults					ATC		11.5 ± 2.8
			healthy adults					PTC		6.3 ± 3.2
Chiu et al. [4]	2000	50	Body	China	17–94	Male and Female	X-ray	ATC	5–22	14.7 ± 3.7
								TPAA	2–18.5	11.5 ± 3.6

*ATC* anterior tibial cortex, *TPAA* tibial proximal anatomical axis, *PTC* posterior tibial cortex

In order to obtain the PTS of normal adults and reduce the measurement errors, the adolescents with unclosed epiphyses and those aged 60 years and older were excluded. This was mainly because of the diversity of epiphyseal morphology with age and because the formation of osteophytes will affect the determination of the tibial plateau tangent. At the same time, knee joints with fractures, bone tumours, osteoarthritis, knee joint surgery, congenital skeletal dysplasia, and knee joint X-rays that did not meet the imaging standards were excluded [19]. Similarly, in order to avoid measurement errors, Kacmaz et al. [18, 19] excluded subjects with unclosed epiphyses and bone disease when conducting PTS studies.

Most previous studies have shown that the PTS differs based on race and region [4, 13, 22, 23, 43–50]. We showed that, even if the same ATC measurement method is used, the measurement results still differ significantly (Table 6). However, in previous studies, when different measurement methods were used, the obtained values still strongly correlated [35, 51]. In this study, the mean PTS in normal adult knee joints in China was 7.68 ± 3.84° (range: 0–21°). Chiu et al. [4] used the ATC method to measure the knee joints of 50 Chinese cadavers and found that the mean PTS value was 14.7 ± 3.7° (range: 5–22°). The findings from their study differed markedly from those in other studies. This is mainly because of the small sample size, and the specific age and sex composition of the included participants.

In the current study, we found that the PTS was significantly related to age and sex. These findings are similar to those reported by Marouane et al. [51, 52]. Using MRI measurements, Hashemi et al. found that the PTS on both the medial and lateral sides were larger in women than in men. However, Kacmaz et al. [18] found that the PTS of men was greater than that of women in a Turkish population. Medda et al. [53] found that there was no significant correlation between the PTS and sex in studies in the Indian population. In the present study, there were no significant differences in the PTS between the left and right sides (*P* > 0.05), similar to the findings reported by Kacmaz et al. [9, 18, 54]. Our study found that PTS differed among different age groups with some regularity, which was similar to the findings of Sun et al. [24]. They studied 1431 subjects aged 0–89 years and found that, the younger the individual, the larger the PTS before age 30 years and the smaller the PTS between the ages of 30 and 59 years. Additionally, the PTS gradually increased after age 60 years (Table 2). PTS differences between the younger and older groups may be related to the regulation of bone growth and degeneration. In general, men develop bones later than women, but as they age, women are more likely than men to develop osteoarthritis of the knee. Zhang et al. [34] reported that, in a group of 60-year-old people in Beijing, the prevalence of knee osteoarthritis, based on X-rays, was 42.8% in women and 21.5% in men. In addition, the PTS has been shown to increase with the onset of osteoarthritis [5, 19]. As a result, in women, who have earlier bone maturation and are more likely to develop degenerative changes and severe osteoarthritis when they are over 40 years, may have higher PTS values than men.

In knee surgery, such as TKA and ACL reconstruction, the PTS plays a vital role in preoperative decision-making and postoperative evaluation [14, 55]. Relevant studies have shown that the PTS will affect the flexion gap, PCL tension, patellofemoral joint contact stress, and knee joint stability after TKA. An excessive PTS will cause the tibia to move forward, the knee joint to become unstable, and the ACL to become tensioned, thereby increasing the risk of ACL injury. Similarly, it will also increase the wear on the polyethylene prosthesis inserted during TKA, resulting in aseptic loosening of the prosthesis. Conversely, a decrease in the PTS will cause the sagittal force line to move forward, increasing the tension on the PCL, causing the prosthesis to sink, narrowing the knee joint space, reducing the range of flexion, and increasing the postoperative stiffness [56]. Therefore, ensuring the accuracy of PTS measurements is key to knee biomechanical balance. Prosthesis manufacturers recommend a PTS of 3–7° during TKA. Okamoto et al. [16] proposed that maintaining the PTS at approximately 5° after TKA might be optimal. The mean PTS in the present study was 7.68 ± 3.84°, which was slightly larger than the value recommended by the prosthesis manufacturer. This implies that, in the Chinese population, the prosthesis manufacturer should adjust the recommended value appropriately. Seo et al. [57] studied 768 patients who underwent TKA and found that a PTS from 3° to − 1° was better, according to the change in PTS that was calculated by subtracting the preoperative PTS from the postoperative PTS. These authors emphasized that patients with a larger preoperative PTS should maintain a larger PTS post-surgery. This will assist the degree of motion of the knee joint after surgery. Kızılgöz et al. [58] emphasized that the PTS measured by lateral X-ray radiographs is very important for determining the risk of ACL injury. Song et al. [11] hypothesized that PTS > 10° was an independent risk factor for tibial anterior displacement and ACL injury. Smith et al. [59] suggested that other factors may also be involved in ACL injury, such as ligament relaxation and hormone levels. Waiwaiole et al. [3, 16, 60–62] found that the PTS is closely related to ACL and PCL injury, and that a PTS significantly larger or smaller than those in individuals of the same sex and age may bring a greater risk of ACL or PCL injury, particularly in athletes and sports participants. The reference values derived in the present study can provide a national basis for the prevention and prediction of ACL or PCL injury. Nha et al. [63] confirmed, through meta-analysis, that PTS increased by 2° after open-wedge HTO, and that appropriate PTS adjustment based on sex and age would improve the postoperative outcomes. The normal range of PTS values among healthy adult knee joints in China identified in this study will benefit the local bone and joint surgeons and can provide guidance to support personalized and precise treatment. We encourage knee prosthesis manufacturers to consider the PTS measurements obtained in the present study for Chinese people, as well as sex and age differences in PTS, in the production of prostheses.

This study had several limitations. China covers a vast territory, including a large population, with various ethnic groups. Thus, our sample was likely not representative of all the individuals within the population. Concurrently, the age range of our study was 25–59 years, which is limited. The PTS was measured using manual methods, and even if the consistency was good, it is likely that there was still some measurement error. Thus, artificial intelligence-assisted computer-based measurement is necessary, both to reduce the workload and to achieve better consistency and standardisation [35]. Due to the limitations associated with X-ray characteristics, it is impossible to distinguish the medial plateau and lateral plateau of the tibia as well as on MRI and CT, and only the average value of the medial plateau and lateral plateau could be obtained. Thus, future studies should include a larger sample size, and AI-assisted measurement software should be trialled.

## Conclusion

This study measured the mean PTS value of healthy adult knee joints in China, using a large population sample, and found that the PTS of healthy Chinese adults differed significantly based on sex and age. Future studies should investigate how marked these differences are based on race and geographic region. The data provided in this study can provide a framework for knee surgery and prosthesis manufacture for this population.

## Data Availability

The datasets used in the current study are available from the corresponding author upon reasonable request and with permission of the Affiliated Hospital of Hangzhou Normal University. However, restrictions apply and the data are not publicly available.

## Abbreviations

ACL: Anterior cruciate ligament
AI: Artificial intelligence
ATC: Anterior tibial cortex
CI: Confidence interval
CT: Computed Tomography
HTO: High tibial osteotomy
ICCs: Intraclass correlation coefficients
MRI: Magnetic resonance imaging
PACS: Picture archiving and communication system
PCL: Posterior cruciate ligament
PTC: Posterior tibial cortex
PTS: Posterior tibial slope
TKA: Total knee arthroplasty
TPAA: Tibial proximal anatomical axis

## References

[1] Giffin JR, Vogrin TM, Zantop T (2004). Effects of increasing tibial slope on the biomechanics of the knee. Am J Sports Med.

[2] Ahmad R, Patel A, Mandalia V (2016). Posterior tibial slope: effect on,and interaction with, knee kinematics. JBJS Rev.

[3] Bernhardson AS, DePhillipo NN, Daney BT, Kennedy MI, Aman ZS, LaPrade RF (2019). Posterior Tibial slope and risk of posterior cruciate ligament injury. Am J Sports Med.

[4] Chiu KY, Zhang SD, Zhang GH (2000). Posterior slope of tibial plateau in Chinese. J Arthroplast.

[5] Han HS, Chang CB, Seong SC, Lee S, Lee MC (2008). Evaluation of anatomic references for tibial sagittal alignment in total knee arthroplasty. Knee Surg Sports Traumatol Arthrosc.

[6] Dean RS, DePhillipo NN, Chahla J, Larson CM, LaPrade RF (2021). Posterior Tibial slope measurements using the anatomic Axis are significantly increased compared with those that use the mechanical Axis. Arthroscopy..

[7] Chambers AW, Wood AR, Kosmopoulos V, Sanchez HB, Wagner RA (2016). Effect of posterior tibial slope on flexion and anterior-posterior tibial translation in posterior cruciate-retaining total knee arthroplasty. J Arthroplast.

[8] Nagamine R, Kawasaki M, Kim KI, Sakai A, Suguro T (2020). The posterior tibial slope is mainly created by the posterior rotation of the tibial condyles. J Orthop Surg (Hong Kong).

[9] Khattak MJ, Umer M, Davis ET, Habib M, Ahmed M (2010). Lower-limb alignment and posterior tibial slope in Pakistanis: a radiographic study. J Orthop Surg (Hong Kong).

[10] Webb JM, Salmon LJ, Leclerc E, Pinczewski LA, Roe JP (2013). Posterior tibial slope and further anterior cruciate ligament injuries in the anterior cruciate ligament-reconstructed patient. Am J Sports Med.

[11] Song GY, Zhang H, Zhang J, Liu X, Xue Z (2018). Greater static anterior tibial subluxation of the lateral compartment after an acute anterior cruciate ligament injury is associated with an increased posterior tibial slope. Am J Sports Med.

[12] Yoo JH, Chang CB, Shin KS, Seong SC, Kim TK (2008). Anatomical references to assess the posterior tibial slope in total knee arthroplasty: a comparison of 5 anatomical axes. J Arthroplast.

[13] Hohmann E, Bryant A, Reaburn P, Tetsworth K (2011). Is there a correlation between posterior tibial slope and non-contact anterior cruciate ligament injuries?. Knee Surg Sports Traumatol Arthrosc.

[14] Kang KT, Koh YG, Son J, Kwon O-R, Lee J-S (2018). A computational simulation study to determine the biomechanical influence of posterior condylar offset and tibial slope in cruciate retaining total knee arthroplasty. Bone Joint Res.

[15] Faschingbauer M, Sgroi M, Juchems M, Reichel H, Kappe T (2014). Can the tibial slope be measured on lateral knee radiographs?. Knee Surg Sports Traumatol Arthrosc.

[16] Okamoto S, Mizu-uchi H, Okazaki K, Hamai S, Nakahara H (2015). Effect of tibial posterior slope on knee kinematics, quadriceps force, and patellofemoral contact force after posterior-stabilized total knee arthroplasty. J Arthroplast.

[17] Kang KT, Koh YG, Son J, Kwon O-R, Lee J-S (2017). Biomechanical effects of posterior condylar offset and posterior tibial slope on quadriceps force and joint contact forces in posterior-stabilized total knee arthroplasty. Biomed Res Int.

[18] Kacmaz IE, Topkaya Y, Basa CD (2020). Posterior tibial slope of the knee measured on X-rays in a Turkish population. Surg Radiol Anat.

[19] Hashemi J, Chandrashekar N, Gill B, Beynnon BD, Slauterbeck JR (2008). The geometry of the tibial plateau and its influence on the biomechanics of the tibiofemoral joint. J Bone Joint Surg Am.

[20] Misir A, Yildiz KI, Kizkapan TB (2019). Wider femoral and mediolaterally narrower tibial components are required for total knee arthroplasty in Turkish patients. Knee Surg Sports Traumatol Arthrosc.

[21] Zhang Y, Wang J, Xiao J, Zhao L, Li Z-H (2014). Measurement and comparison of tibial posterior slope angle in diferent methods based on three-dimensional reconstruction. Knee..

[22] Fan L, Xu T, Li X, Zan P, Li G (2017). Morphologic features of the distal femur and tibia plateau in southeastern Chinese population: a cross-sectional study. Medicine..

[23] Han H, Oh S, Chang CB, Kang SB (2016). Anthropometric difference of the knee on MRI according to gender and age groups. Surg Radiol Anat.

[24] Sun YH, Chen LX, Jiao ZD, Wang L, Zhang RM (2016). Age-related changes of posterior tibial slope and its roles in anterior cruciate ligament injury. Int Surg.

[25] Hohmann E, Tetsworth K, Glatt V, Ngcelwane M, Keough N (2021). Medial and lateral posterior Tibial slope are independent risk factors for noncontact ACL injury in both men and women. Orthop J Sports Med.

[26] de Sousa Filho PGT, Marques AC, Pereira LS, Pigozzo BA, Albuquerque RS (2021). Analysis of posterior tibial slope as risk factor to anterior cruciate ligament tear. Rev Bras Ortop (Sao Paulo).

[27] Schatka I, Weiler A, Jung TM, Walter TC, Gwinner C (2018). High tibial slope correlates with increased posterior tibial translation in healthy knees. Knee Surg Sports Traumatol Arthrosc.

[28] Napier RJ, Garcia E, Devitt BM, Feller JA, Webster KE (2019). Increased radiographic posterior Tibial slope is associated with subsequent injury following revision anterior cruciate ligament reconstruction. Orthop J Sports Med.

[29] Aljuhani W, Qasim SS, Alsalman M (2020). Variability of the posterior tibial slope in saudis: a radiographic study. Cureus.

[30] Aljuaid MO, El-Ghamry OR (2018). Determination of epiphyseal union age in the knee and hand joints bones among the Saudi population in Taif City. Radiol Res Pract.

[31] Washburn SL (1958). Skeletal Age Changes in Young American Males, Analyzed from the Standpoint of Age Identifcation.Tomas W. McKern and T. D. Stewart. Technical Report EP-45, Environmental Protection Research Division, Quartermaster Research and Development Center, U.S. Army, Natick, 1957. viii+ 179 pp., 87 fgs., 52 tables. Am Antiq.

[32] Schaefer MC, Black SM (2005). Comparison of ages of epiphyseal union in north American and Bosnian skeletal material. J Forensic Sci.

[33] O'Connor JE, Bogue C, Spence LD, Last J (2008). A method to establish the relationship between chronological age and stage of union from radiographic assessment of epiphyseal fusion at the knee: an Irish population study. J Anat.

[34] Zhang Y, Xu L, Nevitt MC, Aliabadi P, Yu W, Qin M (2001). Comparison of the prevalence of knee osteoarthritis between the elderly Chinese population in Beijing and whites in the United States: the Beijing osteoarthritis study. Arthritis Rheum.

[35] Utzschneider S, Goettinger M, Weber P (2011). Development and validation of a new method for the radiologic measurement of the tibial slope. Knee Surg Sports Trawnatol Arthrosc.

[36] Wang S, Xiao Z, Lu Y, Zhang Z, Lv F (2021). Radiographic optimization of the lateral position of the knee joint aided by CT images and the maximum intensity projection technique. J Orthop Surg Res.

[37] Green DW, Sidharthan S, Schlichte LM, Aitchison AH, Mintz DN. Increased posterior Tibial slope in patients with Osgood-Schlatter disease: A new association. Am J Sports Med. 2020;(3):642–6. 10.1177/0363546519899894 Epub 2020 Jan 31. PMID: 32004085. 10.1177/0363546519899894 32004085

[38] Yuan HS, Xu WJ (2017). Graphic guidelines for imaging of the musculoskeletal system.

[39] Kessler MA, Burkart A, Martinek V (2003). Development of a 3-dimensional method to determine the tibial slope with multislice-CT. Z Orthop Hire Grenzgeb.

[40] Bao L, Rong S, Shi Z, Wang J, Zhang Y (2021). Measurement of femoral posterior condylar offset and posterior tibial slope in normal knees based on 3D reconstruction. BMC Musculoskelet Disord.

[41] Kim KH, Bin SI, Kim JM (2012). The correlation between posterior Tibial slope and maximal angle of flexion after Total knee Arthroplasty. Knee Surg Relat Res.

[42] Oka S, Matsumoto T, Muratsu H, Kubo S, Matsushita T (2014). The influence of the tibial slope on intra-operative soft tissue balance in cruciate-retaining and posterior-stabilized total knee arthroplasty. Knee Surg Sports Traumatol Arthrosc.

[43] Ho WP, Cheng CK, Liau JJ (2006). Morphometrical measurements of resected surface of femurs in Chinese knees: correlation to the sizing of current femoral implants. Knee..

[44] Faschingbauer M (2021). Editorial commentary: posterior Tibial slope: the "unknown size" of the knee joint. Arthroscopy..

[45] De Boer JJ, Blankevoort L, Kingma I, Vorster W (2009). In vitro study of inter-individual variation in posterior slope in the knee joint. Clin Biomech (Bristol, Avon).

[46] Noyes FR, Goebel SX, West J (2005). Opening wedge tibial osteotomy: the 2-triangle method to correct axial alignment and tibial slope. Am J Sports Med.

[47] Zeng C, Gao SG, Wei J, Yang TB, Cheng L (2013). The infuence of the intercondylar notch dimensions on injury of the anterior cruciate ligament: a meta-analysis. Knee Surg Sports Traumatol Arthrosc.

[48] Vaidya SV, Ranawat CS, Aroojis A, Laud NS (2000). Anthropometric measurements to design total knee prostheses for the Indian population. J Arthroplast.

[49] Kuwano T, Urabe K, Miura H, Nagamine R, Matsuda S (2005). Importance of the lateral anatomic tibial slope as a guide to the tibial cut in total knee arthroplasty in Japanese patients. J Orthop Sci.

[50] Winkler PW, Godshaw BM, Karlsson J, Getgood AMJ, Musahl V (2021). Posterior tibial slope: the fingerprint of the tibial bone. Knee Surg Sports Traumatol Arthrosc.

[51] Marouane H, Shirazi-Adl A, Adouni M, Hashemi J (2014). Steeper posterior tibial slope markedly increases ACL force in both active gait and passive knee joint under compression. J Biomech.

[52] Bisicchia S, Scordo GM, Prins J, Tudisco C (2017). Do ethnicity and gender influence posterior tibial slope?. J Orthop Traumatol.

[53] Medda S, Kundu R, Sengupta S, Pal AK (2017). Anatomical variation of posterior slope of tibial plateau in adult eastern Indian population. Indian J Orthop.

[54] Aljuhani WS, Qasim SS, Alrasheed A, Altwalah J, Alsalman MJ (2021). The effect of gender, age, and body mass index on the medial and lateral posterior tibial slopes: a magnetic resonance imaging study. Knee Surg Relat Res.

[55] Kang KT, Koh YG, Son J, Kwon OR, Lee JS (2018). Infuence of increased posterior tibial slope in total knee arthroplasty on knee joint biomechanics: a computational simulation study. J Arthroplast.

[56] Okazaki K, Tashiro Y, Mizu-uchi H, Hamai S, Doi T (2014). Influence of the posterior tibial slope on the flexion gap in total knee arthroplasty. Knee.

[57] Seo SS, Kim CW, Kim JH, Min YK (2013). Clinical results associated with changes of posterior tibial slope in total knee arthroplasty. Knee Surg Relat Res.

[58] Kızılgöz V, Sivrioğlu AK, Ulusoy GR, Yıldız K, Aydın H (2019). Posterior tibial slope measurement on lateral knee radiographs as a risk factor of anterior cruciate ligament injury: a cross-sectional study. Radiography (Lond).

[59] Smith HC, Vacek P, Johnson RJ, Slauterbeck JR, Hashemi J (2012). Risk factors for anterior cruciate ligament injury: a review of the literature. Part 1: neuromuscular and anatomic risk. Sports. Health..

[60] Waiwaiole A, Gurbani A, Motamedi K, Seeger L, Sim MS, Nwajuaku P, Hame SL (2016). Relationship of ACL injury and posterior Tibial slope with patient age, sex, and race. Orthop J Sports Med.

[61] Sessa P, Fioravanti G, Giannicola G, Cinotti G (2015). The risk of sacrificing the PCL in cruciate retaining total knee arthroplasty and the relationship to the sagittal inclination of the tibial plateau. Knee..

[62] Bellemans J, Robijns F, Duerinckx J, Banks S, Vandenneucker H (2005). The influence of tibial slope on maximal flexion after total knee arthroplasty. Knee Surg Sports Traumatol Arthrosc.

[63] Nha KW, Kim HJ, Ahn HS, Lee DH (2016). Change in posterior Tibial slope after open-wedge and closed-wedge high Tibial osteotomy: a meta-analysis. Am J Sports Med.

